# The connection between lymphangiogenic signalling and prostaglandin biology: A missing link in the metastatic pathway

**DOI:** 10.18632/oncotarget.593

**Published:** 2012-08-19

**Authors:** Tara Karnezis, Ramin Shayan, Stephen Fox, Marc G. Achen, Steven A. Stacker

**Affiliations:** ^1^ Peter MacCallum Cancer Centre, Locked Bag 1, A'Beckett Street, East Melbourne, Victoria, Australia; ^2^ Department of Surgery, Royal Melbourne Hospital, University of Melbourne, Victoria, Australia; ^3^ Jack Brockhoff Reconstructive Plastic Surgery Research Unit, Royal Melbourne Hospital and Department of Anatomy, Faculty of Medicine, Dentistry and Health Sciences, University of Melbourne, Victoria, Australia; ^4^ Sir Peter MacCallum Department of Oncology, University of Melbourne, Parkville, Australia

**Keywords:** Cancer, Metastasis, VEGF-D, Lymphatics, Lymphangiogenesis, Angiogenesis, Prostaglandin, PGDH, COX-1, COX-2, NSAID, Aspirin

## Abstract

Substantial evidence supports important independent roles for lymphangiogenic growth factor signaling and prostaglandins in the metastatic spread of cancer. The significance of the lymphangiogenic growth factors, vascular endothelial growth factor (VEGF)-C and VEGF-D, is well established in animal models of metastasis, and a strong correlation exits between an increase in expression of VEGF-C and VEGF-D, and metastatic spread in various solid human cancers. Similarly, key enzymes that control the production of prostaglandins, cyclooxygenases (COX-1 and COX-2, prototypic targets of Non-steroidal anti-inflammatory drugs (NSAIDs)), are frequently over-expressed or de-regulated in the progression of cancer. Recent data have suggested an intersection of lymphangiogenic growth factor signaling and the prostaglandin pathways in the control of metastatic spread via the lymphatic vasculature. Furthermore, this correlates with current clinical data showing that some NSAIDs enhance the survival of cancer patients through reducing metastasis. Here, we discuss the potential biochemical and cellular basis for such anti-cancer effects of NSAIDs through the prostaglandin and VEGF signaling pathways.

## INTRODUCTION

The lethality of many solid tumors is primarily associated with the ability to spread to distant organs in a process known as metastasis [1]. Historically, two theories have been proposed to explain metastatic spread. In Paget's “seed and soil” theory (1889), tumor cells have a propensity to colonize certain organs with the appropriate cellular and molecular milieu to encourage tumor cell survival and growth, whereas Ewing (1928) proposed that metastasis relies on “anatomical and mechanical” routes that utilize the blood and lymphatic vasculature which have defined distributions and anatomical locations in the body[2, 3]. It is well recognized that tumor cells can metastasize from the primary tumor into surrounding tissue (local spread), into blood and lymphatic vessels and from there to regional lymph nodes and major organ systems (e.g. brain, bone, liver or lung), and it is therefore likely that the metastatic pattern of solid tumors combines both the “seed and soil” and “anatomical and mechanical” theories.

The complex multistep process of metastasis involves local invasion of cancer cells followed by intravasation of cancer cells into blood and/or lymphatic vessels; trafficking of cancer cells through these vessels; extravasation to the lymph node or distant organs; formation of micrometastases consisting of small cancer nodules, and finally the formation of macrometastases. The complexity of these processes indicates that they are likely to depend on a multitude of signalling networks [4]. The metastatic potential of cancer cells depends on factors such as the intrinsic genetic properties of the tumor itself that enables tumor cells to survive and proliferate. In addition, the tumor microenvironment, which consists of cells such as endothelial cells (ECs), fibroblasts, macrophages and other immune cells, mesencheymal stem cells and the extracellular matrix, is a key determinant of metastatic potential [4]. Tumor-stromal cell interactions during the course of metastatic disease can induce the production of VEGF family members and other signalling mediators, such as prostaglandins (PGs), which can lead to alterations of the cells comprising the local tumor microenvironment, and to other cell types beyond the tumor mass promoting the growth and dissipation of tumor cells [5, 6].

Because cancer cell migration through connective tissue is arduous and slow, tumor cells are able to traffic more quickly and efficiently via blood or lymphatic vessels [7, 8]. The primary roles of the lymphatic vasculature are absorption of fluid from surrounding tissue, absorption of dietary fat and immune cell surveillance. However, in cancer, lymphatics can provide a conduit for tumor cell dissemination [9]. Tumor and tumor-stromal cells can stimulate nearby lymphatic endothelial cell recruitment, proliferation, migration and tubule formation resulting in new lymphatic vessels within and immediately around solid tumors and draining lymph nodes in a process known as lymphangiogenesis. A large proportion of metastatic tumor cells follow the pattern of spread via the lymphatics (i.e. lymphogenous spread), beginning at the primary tumor, spreading through the lymphatic vessels, to the sentinel lymph nodes [10, 11]. Clinical studies on patients with breast, colon, prostate cervical, ovarian, head and neck cancers and melanomas, have revealed that an early sign of cancer spread is the detection of tumor cells in the draining sentinel lymph node, with spread to lymph nodes considered an important initial step of metastasis and a key parameter in staging and treating human cancer [12]. The lymphatic system is therefore a key route for tumor spread [13] [14] [15].

Members of the VEGF family promote the formation of tumor-associated blood and lymphatic vessels and influence the growth and spread of tumor cells. In particular, two members of this family, VEGF-C and VEGF-D, have been identified as lymphangiogenic factors [16-18] acting via specific VEGF receptors expressed on lymphatic endothelial cells [19]. The VEGF-C/VEGF-D signaling axis is believed to play a pivotal role in the control of lymphangiogenesis during disease making it an attractive target for therapeutic intervention [20].

Recently, preclinical and clinical trials using Non-Steroidal Anti-Inflammatory Drugs (NSAIDs) such as aspirin, indomethacin, etodolac, sulindac and ibuprofen- typically used to treat inflammation - have been shown to reduce cancer incidence, metastasis and ultimately overall cancer morbidity suggesting that NSAIDs may be used in the treatment of metastatic disease, potentially linking the role of prostaglandins and lymphangiogenesis during metastasis [21-24] [6, 25].

In this review, we will describe the nature of the lymphatic system in the context of metastasis and explore the role of VEGF-C/VEGF-D and prostaglandin signaling pathways in lymphogenous spread, with a view to the clinical benefit of NSAIDs to target lymphatic vessels during metastatic disease.

### The lymphatic vasculature during cancer

Tumor cells can enter the lymphatic system by invading pre-existing lymphatic vessels in the tumor periphery or by eliciting tumor associated-lymphangiogenesis [14, 26]. Lymphangiogenesis is driven primarily by lymphangiogenic growth factors, such as VEGF-C and VEGF-D, and is a multi-faceted process involving aspects of endothelial cell proliferation, migration, adhesion, sprouting, tubule and lumen formation, dilation and maturation, all of which act in concert to generate functional lymphatic vessels [9].

The lymphatic system is composed of a hierarchy of vessels, beginning within the superficial dermis of the skin as initial lymphatic vessels (also known as lymphatic capillaries) that merge into the pre-collecting lymphatic vessels, located in the deep dermis, which in turn drain into the collecting lymphatics, located in the subcutaneous tissue [12, 27-29]. It is becoming apparent that lymphatic vessel subtypes undergo changes in response to lymphangiogenic factors during the course of metastasis. An important clue to the responsiveness of lymphatic vessel subtypes to lymphangiogenic growth factors was described in animal models of lymphogenous spread in which proliferation of nearby initial lymphatics increased the number of lymphatic vessels in and around the tumor, resulting in an increase in the contact surface area between the invading cancer cells and the lymphatic endothelium [30].

Lymphatic vessel density has been shown to be prognostically significant in several human malignancies [13, 31]. In addition to lymphatic vessel density, VEGF-C and VEGF-D have been demonstrated to be important prognostic indicators in several human tumors such as malignant melanoma where it was shown that peritumoral lymphatic vessel density and lymphangiogenic growth factor expression were important in determining which tumors metastasize to regional lymph nodes [32]. Increased lymphatic vessel density in tumors is also associated with increased metastasis to lymph nodes [33]. However, it has been debated whether intratumoral lymphatic vessel density has any prognostic value because, unlike the peritumoral lymphatics, intratumoral lymphatic vessels have been proposed to be non-functional [30] [34].

For tumor cells to spread via the lymphatic vasculature to sentinel lymph nodes, the lymphatic vessels are ‘prepared’ by the tumor cells for effective invasion and spread. Activation of the lymphatic endothelium by tumor and tumor-associated stromal cell secreted lymphangiogenic factors may change the adhesive properties of the lymphatic endothelium, promoting tumor cell-lymphatic endothelial cell interactions or leading to an increase in lymphatic vessel size, thus facilitating entry of tumor cells into the initial lymphatics [35]. In addition, VEGF-C or VEGF-D secreted by tumor cells may increase vascular permeability or have important effects on the tumor interstitial-fluid pressure which may promote tumor-cell entry into the lymphatics [13] [36, 37]. Lymphangiogenic growth factors produced by tumor cells and tumor-associated stromal cells stimulate growth and dilation of peritumoral lymphatic vessels surrounding the primary tumor [38]. Similarly, VEGF-C or VEGF-D were also shown to promote morphological changes in collecting lymphatic vessels draining the primary tumor mass [6]. It was observed that collecting lymphatic vessels dilated during the course of VEGF-C or VEGF-D driven metastasis [6, 39, 40]. In the case of VEGF-C driven metastatic models, dilation of the collecting lymphatic vessels resulted in increased functional flow and a concomitant increase in the number of metastatic cells reaching the regional lymph nodes [6, 39, 40]. The mechanism(s) by which lymphangiogenic growth factors exert their biological effects are only beginning to emerge [9]. Understanding the interplay between the lymphangiogenic VEGF family members and other signaling pathways will be important for the design of more effective and specific therapeutic agents that target the lymphogenous spread of cancer.

### Molecular regulation of tumor lymphangiogenesis: the VEGF-C/VEGF-D signaling axis

Vascular endothelial growth factors (VEGFs) are critical regulators of blood and lymphatic vessel formation during both development and disease. In mammals, five VEGF ligands (VEGF-A, -B, -C, -D and placenta growth factor (PlGF)) have been identified with structurally related proteins encoded by parapoxvirus (VEGF-E) and found in snake venom (VEGF-F; for other non-VEGF proteins that induce lymphangiogenesis refer to [11]). VEGFs mediate their effects by binding in an overlapping pattern to three receptor tyrosine kinases; VEGF receptor-1 (VEGFR-1), -2 and -3. While VEGF-A (also known as VEGF or vascular permeability factor (VPF)) has been identified as the dominant angiogenic factor in many human and experimental murine cancers, acting via VEGFR-1 and VEGFR-2 expressed on endothelial cells lining blood vessels[41-43], the key VEGF family members driving lymphangiogenesis are VEGF-C and VEGF-D (although VEGF-A can also induce lymphangiogenesis) [44-47]. In pre-clinical animal models, VEGF-C and VEGF-D increase tumor-associated lymphangiogenesis and lymph node and distant organ metastasis [48-50], and there is a strong correlation between VEGF-C and/or VEGF-D expression and metastatic spread and poor patient outcome in a variety of human cancers [49-52].

VEGF-C and VEGF-D are secreted homodimeric glycoproteins with a central VEGF homology domain (VHD) containing receptor binding sites for VEGFR-2 and VEGFR-3. The VHD is flanked by N- and C-terminal propeptides which can be proteolytically processed to produce mature forms, consisting of the VHD, with high affinity for VEGFR-2 and VEGFR-3 [16, 53-61]. The capacity of VEGF-C and VEGF-D to promote tumor angiogenesis and lymphangiogenesis, as well as tumor growth and/or spread, was blocked when the proteolytic cleavage sites were abolished by mutation, demonstrating that processing of these proteins is important for their biological effects in cancer[62] [63, 64].

VEGF-C and VEGF-D mediate their biological effects by binding VEGFR-2 or -3 expressed on the surface of blood vascular and lymphatic endothelial cells [16, 53, 65] [66] [19, 39, 67, 68]. In response to binding these growth factors, VEGFR-3 forms homodimers or heterodimers with VEGFR-2 which leads to activation of the receptor-tyrosine kinase activity inducing auto-phosphorylation of the receptors; phosphorylated receptors then recruit interacting proteins and, in the case of lymphatics, induce specific signaling cascades to activate various aspects of lymphatic endothelial cell function and ultimately, lymphatic vessel formation [66, 69]. VEGFR-3 expression was shown to correlate with lymphatic metastasis in some prevalent human cancers[52]. For example, it has been reported that the presence of VEGF-D and VEGFR-3 in endometrial carcinoma may be a prognostic indicator for lymph node spread [70]. Further, VEGFR-3 expression by lymphatic endothelial cells in human prostate cancer is thought to be important for metastatic spread of tumor cells to the lymph nodes [71]. In addition to its role in tumor lymphangiogenesis, VEGFR-3 is important for blood vessel remodeling, lymphangiogenesis and angiogenesis during embryonic development and in other biological settings [18, 68, 72-74].

VEGF-C has been shown to stimulate migration of endothelial cells, induce vascular permeability and endothelial cell proliferation, induce intercellular gaps between lymphatic endothelial cells that facilitate entry of tumor cells into the lumen of lymphatic vessels, and promote lymphatic vessel enlargement that increases lymph flow and trafficking of tumor cells to lymph nodes [16, 40, 61, 75]. These effects are mediated predominately by VEGFR-3 signaling, although VEGFR-2 can play a role.

VEGF-C and VEGF-D signaling via VEGFR-3 can also be modulated by co-receptors such as the neuropilins [62, 76]. Neuropilin-2 is important in lymphatic development as has been shown by the phenotype of *Neuropilin-2^−/−^* mice, which fail to form normal lymphatic vessels and capillaries [77].

It is becoming apparent that other signaling pathways operate within lymphatic endothelial cells, or cells associated with lymphatic vessels, that are likely to contribute to lymphangiogenesis [78-82] [9, 83]. For example, tumor-associated fibroblasts produce high levels of hyaluronan within the tumor stroma stimulating cancer cells to secrete lymphangiogenic proteins [84]. Recently, cross-talk between the lymphangiogenic growth factors VEGF-C and VEGF-D, and prostaglandin signaling pathways, has been demonstrated to facilitate metastasis suggesting that there is a molecular link between the VEGFs and prostaglandin signaling axes [85, 86]

### Prostaglandins, COX and lymphangiogenesis

Isolation of endothelial cells from normal tissues and from blood or lymphatic vessels exposed to angiogenic or lymphangiogenic growth factors has identified molecular signatures that are important during tumor-associated angiogenesis and lymphangiogenesis [87, 88]. Recently, it was revealed that lymphangiogenic growth factors influenced the expression of key prostaglandin (PG) pathway genes in lymphatic endothelium, suggesting that there is cross-talk between PG and VEGF-C and VEGF-D signaling [6]. This is consistent with the elevated levels of inflammatory mediators, such as PGs, that have been reported in human cancer [89].

PGs are a class of bioactive lipids that are produced in a wide variety of human tissues and have a central role in development, tissue homeostasis, inflammation and cancer progression [90, 91]. PGs are involved in a range of cellular functions, and have been shown to be potent inducers of vasodilation thereby acting as modulators of vascular tone [90]. The importance of PG-mediated dilation during metastasis was demonstrated in an animal model of lymphogenous spread, in which it was shown that the lymphangiogenic factors VEGF-C and VEGF-D were able to induce dilation of collecting lymphatic vessels draining the primary tumor mass, leading to increased metastatic load in the sentinel lymph node [6].

Cyclooxygenases (COXs) are the rate-limiting enzymes that catalyze the conversion of arachidonic acid to PGs in tissues. Two COX isoforms have been identified with distinct functions and tissue distributions; COX-1 is constitutively expressed in most tissues and is important in maintaining basal PG levels important for tissue homeostasis, whereas COX-2 can be induced in most tissues producing pro-inflammatory PGs during inflammation and tumorigenic settings. A third enzyme, COX-3, has been identified but is considered a variant of COX-1 that arises due to differential RNA splicing [92]. COX-2 is up-regulated in many types of cancers including lung, colon, breast, pancreas and head and neck cancers [93-101]. The biochemical activity of COX-2 is balanced by the prostaglandin degrading enzyme 15-hydroxyprostaglandin dehydrogenase (PGDH), which catalyzes NAD+ dependent conversion of the prostaglandin 15-OH to an inactive 15-keto group within the cytoplasm of cells [102]. Interestingly, the expression of PGDH was shown to be abrogated in animal models of colon cancer and thus, PGDH is considered a tumor suppressor [103-106]. In addition, the levels of PGs can be regulated by PG transporter proteins (PGT) and multidrug resistance associated protein 4 (MRP4)[107]. The production of PGs is therefore balanced by degradation, and factors that influence the activity or expression of either biosynthetic and/or degrading enzymes by genetic and/or pharmacological intervention are likely to lead to imbalances in the levels of PGs produced by various tissues, in turn affecting the growth and metastatic potential of tumors.

**Figure 1:**
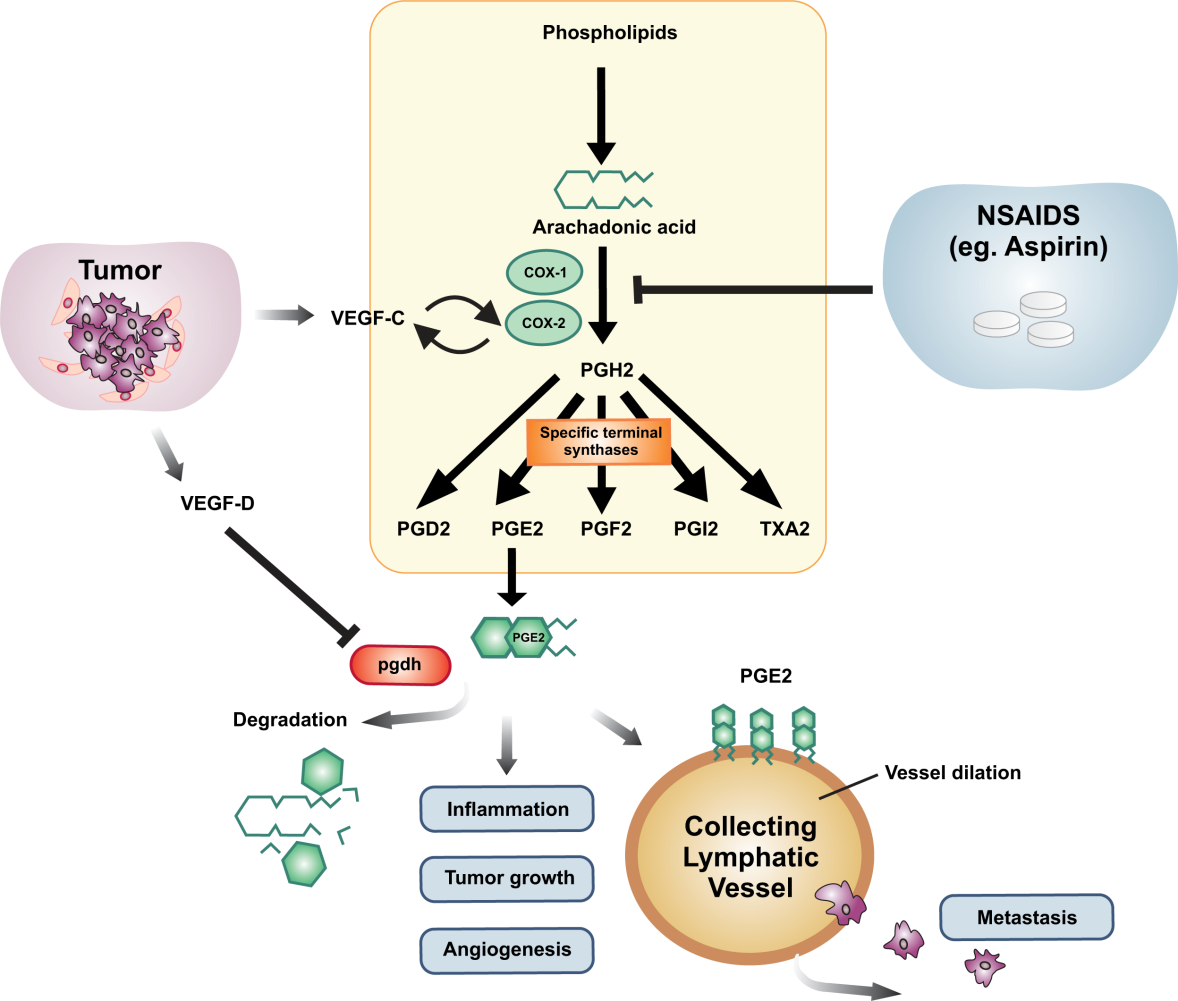
Schematic representation of the interplay between the lymphangiogenic growth factors, the prostaglandin pathway and cancer metastasis Prostaglandins are synthesized from Arachidonic acid and this is catalyzed by the enzymes cyclooxygenase-1 (COX-1) and COX-2. The intermediate PGH_2_ gives rise to PGD_2_, PGE_2_, PGF_2_, PGI_2_ and TXA_2_ via specific terminal synthases. PGE_2_ is the form of prostaglandin that is associated with inflammation, tumor growth and angiogenesis. PGE_2_ is degraded by the enzyme, 15-hydroxyprostaglandin dehydrogenase (*pgdh*). Tumors and their associated stroma secrete a number of angiogenic and lymphangiogenic growth factors that act on blood and lymphatic vessels to promote cancer. Members of the VEGF family of growth factors, VEGF-C and VEGF-D, are secreted by tumors and act on small lymphatic vessels in and around the primary tumor, as well as on larger collecting lymphatic vessels which carry metastatic tumor cells from the primary tumor to regional lymph nodes. VEGF-D has been shown to cause dilation of collecting lymphatic vessels via down-regulation of the *pgdh* gene leading to increased metastasis. This results in reduced PGE_2_ degradation causing dilation of collecting lymphatic vessels expressing appropriate PGE2 receptors. VEGF-C has been reported to induce expression of COX-2 and vice versa. Therapeutically, this can be inhibited by antagonists of the COX pathway, which can reduce the amount of PGE_2_ being synthesized. Members of the NSAID family, such as Aspirin, can antagonize COX-mediated production of PGE_2_.

COX enzymes catalyze the conversion of arachidonic acid to prostaglandin H2 which serves as the precursor for many PGs formed by the action of specialized PG synthases, which can become up-regulated in some cancers [108-110]. PGs formed in this pathway include PGE_2_, PGD_2_, PGF_2α_, PG I_2_ and thromboxane-A2 [111] (See Figure 1). Neoplastic tissues, such as human colon cancers, contain high concentrations of PGs, with the pro-tumorigenic effects of COX-2 believed to be largely attributed to its role in producing PGE2 [112]. COX-2 expression is associated with poor patient prognosis and survival and although COX-2 might be the key player in PG production during tumorigenesis, evidence also implicates COX-1 as a causal agent, likely due to its influence on PGE2 concentration [113-115].

PGE2 exerts its effects by engaging members of the G-coupled superfamily of receptors, EP1-4 [116]. Upon ligation of the cognate receptors, signal transduction cascades are activated to modulate intracellular levels of cAMP and Ca^++^ that impact on various aspects of cell biology such as proliferation, adhesion, invasion, motility, cell morphology and survival of both tumor cells and surrounding tumor-associated stromal cells [117]. Tumor studies conducted in EP1-4 receptor knockout animals or using pharmacological inhibition revealed that these receptors have a role in promoting tumorigenesis, angiogenesis and lymphangiogenesis in various tumor settings and therefore provide attractive targets for therapeutic intervention [118, 119, 120, 121]. In addition, some PGs, including PGE2, can bind to nuclear receptors known as peroxisome proliferator-activated receptor proteins (PPARs) leading to activation of the APC-β catenin/Wnt pathway [122, 123].

### Intersection of prostaglandin and VEGF-C/VEGF-D signaling pathways to regulate metastasis

Several clinical studies have shown a correlation between the level of COX-2 expression and the extent of angiogenesis in breast, endometrial and gastric cancers [124-126]. Clues to the link between VEGF family members and PG signaling pathways were first observed in mouse models of colon cancer, in which a correlation between COX-2, VEGF-A expression and angiogenesis was reported [127]. For instance, COX-2 over-expression in tumor cells stimulated production of VEGF-A which caused blood vascular endothelial cell migration and tube formation [128]. In addition to inducing VEGF-A expression by tumor cells, PG signaling in stromal cells also contributed to angiogenesis. In both benign and malignant colon cancers, tumor-associated stromal expression of COX-2 resulted in elevated levels of PGE2 that stimulated the PG receptor, EP-2, followed by induction of VEGF-A production that promoted tumor angiogenesis [129]. Similarly, stromal PGE2-EP3 signaling is required for VEGF-A expression and tumor-associated angiogenesis in lung carcinoma whereas PGE2-EP2 signalling enhanced motility and survival of blood vascular endothelial cells, as well as angiogenesis, in a model of breast cancer [121, 130]. Evidence suggests that PGE2 is the signaling mediator regulating the production of VEGF-A, and therefore angiogenesis. It was shown that angiogenic blockade achieved with a COX-2 inhibitor or by administration of a PGE2 monoclonal antibody could be reversed by treatment with PGE2 [112, 131, 132].

In contrast to the well-established effect of the COX-2 and PG pathway on angiogenesis, effects on lymphangiogenesis are only beginning emerge. Clinical and histopathological studies have revealed a correlation between COX-2 expression, lymphatic vessel density and lymph node metastasis in several human malignancies [133-136]. Much of the current data suggests that the PG pathway can influence lymphatic vessel density, and therefore lymph node metastasis, by regulating the levels of VEGF-C and VEGF-D produced within the tumor and the tumor microenvironment [85, 137, 138]. PGE2 was shown to be the mediator for this effect acting via different EP receptors expressed on the surface of various tumor-associated stromal cells. EP3 signaling has been shown to contribute to tumor lymphangiogenesis [139]. For instance, EP3 activation caused an increase in VEGF-C and VEGF-D secretion by cultured macrophages whereas EP4 activation elevated VEGF-C secretion by tumor-associated macrophages and VEGF-D secretion by tumor-associated fibroblasts leading to enhanced lymphangiogenesis within the primary tumor [138]. In addition to VEGF-C regulation via EP-3, COX-2 was shown to regulate VEGF-C production and lymphangiogenesis in human lung adenocarcinoma via the EP-1/Src/HER-2/Neu signaling pathway [140].

Interestingly, recent studies have indicated regulation of tumor lymphangiogenesis downstream of COX-2 within lymphatic endothelial cells [6]. It was discovered that the degrading arm of the PG pathway, involving the key PGE2 degrading enzyme, PGDH, is regulated by the VEGF-D/VEGFR-2/VEGFR-3 signaling pathway. Hence, expression of VEGF-D in a mouse tumor model led to a rise in tissue PGE2 levels[6]. In this study, the levels of PGE2, in part, caused an alteration in the morphological characteristics of collecting lymphatic endothelial cells, enhancing dilation of the collecting lymphatic vessel draining the primary tumor. This was associated with increased tumor load in the sentinel lymph node. The dilation observed is in keeping with PGDH-null mice that have increased PGE2 levels as well as a patent ductus ateriosus [141]. A key effect of PGE2 and VEGF-D in the setting of cancer is to regulate dilation or patency of collecting lymphatic vessels draining the tumor which is a mechanism for enhancing metastasis.

The interaction between the PG and VEGF-C/VEGF-D pathways has led investigators to define the impact of NSAIDs, the prototypic inhibitors of COX-2, on metastatic disease. It has been shown that regular intake of NSAIDs reduces the incidence of cancers but the significance on metastasis is only beginning to emerge [24, 111].

### NSAIDs as a therapeutic option for treatment of metastatic disease

Therapeutic approaches for targeting the signaling induced by soluble growth factors, such as VEGF-C and VEGF-D, include monoclonal antibodies to the growth factors or their receptors, soluble receptors and small molecule inhibitors targeting the tyrosine kinase activity of the receptors. These approaches have achieved various degrees of success in pre-clinical models and clinical studies [11, 14, 142]. Such approaches targeting VEGF-C/VEGF-D/VEGFR-3 have the potential to block the lymphogenous spread of cancer as well as tumor growth and hematogenous spread given this signaling axis can contribute to tumor angiogenesis [11, 14]. Regardless of recent advances in chemotherapy, radiotherapy and surgery, the prognosis of many cancers remains poor, highlighting the need for new or additional drugs as anti-metastatic therapies.

NSAIDs are a diverse group of similarly acting drugs that have been traditionally used to treat inflammatory disease, such as rheumatoid arthritis. As such, they have strong effects on inflammation and have found uses during analgesia and as anti-pyretics [143, 144]. NSAIDs can be selective inhibitors against COX-1 (e.g. Ketoprofen) or COX-2 (e.g. Celecoxib, Etodolac, Rofecoxib) or non-selective, inhibiting both COX enzymes (e.g. Aspirin, Naproxen, Ibuprofen) [144]. It should be noted that some NSAIDs mediate their effects independent of COX enzymes [145] [146]. Upon binding to their respective targets, NSAIDs inhibit PG synthesis which has profound effects on cell proliferation, migration, apoptosis and angiogenesis, key features of tumorigenesis [144].

Given the involvement of PGs in the progression and spread of cancer, the potential role of aspirin in cancer prevention is worthy of investigation but has been only recently explored as there was concern about the potential risk of aspirin-induced bleeding, predominantly in the upper gastrointestinal tract [111]. However, compelling epidemiological studies have revealed that aspirin, which inhibits COX-1 at low concentrations and is a non-selective COX-1/COX-2 inhibitor at high concentrations, can reduce the overall incidence and mortality of colon cancer when administered at daily low doses [111, 147]; [22-24]. Interestingly, further clinical trials revealed that treatment with NSAIDs, such as Aspirin, can reduce tumor spread in breast and prostate cancer patients, yet the precise anti-metastatic mechanism(s) was unclear [148-150] [151] [111];[23, 24].

The effects of NSAIDs, including Aspirin, Celecoxib, Rofecoxib, SC-5600, Etodolac and Nimesulide, was assessed on both lymphangiogenic growth factor expression and the lymphatic vasculature in pre-clinical models of metastatic disease. *In vitro* treatment of breast and esophageal tumor cell lines with nimesulide, diclofenac, rofecoxib and SC-5600 caused a down-regulation of VEGF-C expression [152, 153]. In a model of gastric cancer, it was shown that treatment with a COX-2 inhibitor, Etodolac, reduced lymphangiogenesis that in turn led to a decrease in metastasis to sentinel lymph nodes. The authors also showed that this was in part attributed to a reduction in the level of VEGF-C secreted from surrounding macrophages [85] [85]. Likewise, treatment of a mouse model of lung cancer with Celecoxib, a COX-2 selective inhibitor, caused a reduction in VEGF-C and VEGFR-3 expression with a subsequent decrease in lymphatic vessel density and metastasis [139, 154]. More recently, a mechanism for the effects of NSAIDs on lymphogenous spread was proposed. Etodolac was shown to reduce the metastatic load, in both regional lymph nodes and distant organs, in models of VEGF-D driven lymphogenous spread by reversing the morphological changes in collecting lymphatic vessels [6]. Based on collective data from pre-clinical animal models, the anti-metastatic effects of NSAIDs observed in clinical trials may be explained, in part, due to their ability to suppress or inhibit tumor-associated lymphangiogenesis, a necessary component of lymphogenous spread [6]. While most clinical trials involving NSAIDs have emphasized tumor growth, mortality and distant organ metastasis, future clinical trials evaluating the efficacy of NSAIDS on tumor metastasis should also focus on clinical evaluations of tumor lymphatics and lymph node metastasis.

### SUMMARY AND FUTURE DIRECTIONS

The active role of lymphatic vessels in lymphogenous spread and the identification of the key lymphangiogenic growth factors, VEGF-C and VEGF-D, have led to novel therapeutic approaches that target the VEGF-C/VEGF-D signaling axis including neutralizing antibodies, soluble receptors and small molecule inhibitors. Emerging evidence from histopathological, genetic and clinical analyses has revealed that the VEGF-C/VEGF-D and PG signaling pathways intersect, adding to the number of pathways that make up the total signaling necessary to establish lymphogenous spread. This knowledge emphasizes the need to understand the interplay between signaling networks which will assist in identification of biomarkers, development of novel therapeutic agents or use of existing agents that target multiple pathways for a more integrative approach. The use of multiple agents in chemotherapy can facilitate the efficacy of these drugs at lower doses due to synergistic effects and because known biological features of cancer such as proliferation, inflammation, angiogenesis and lymphangiogenesis may be targeted simultaneously. The proposed effects of NSAIDs on lymphangiogenesis and lymphatic vessel dilation may have therapeutic implications in chemoprevention, adjuvant chemotherapy and treatment of metastatic disease.

An exciting new approach for the treatment of metastatic disease is referred to as personalized medicine. This approach relies on the molecular profiling of individual cancers and the analysis of specific biomarkers. This idea is supported by recent genetic studies on colon cancers that revealed those patients with reduced COX expression and/or polymorphisms in both COX and PGDH genes may not benefit from NSAID treatment [155-157]. Likewise, screening the same patients for polymorphisms in components of the VEGF-C/VEGF-D signalling pathway may assist in the type of anti-lymphangiogenic therapy administered to improve patient outcome [158, 159]. The interaction of the VEGF-C/VEGF-D and PG signalling axes may provide additional biomarkers such as circulating levels of VEGF-C, VEGF-D and PG for predicting which patients respond to anti-metastatic therapies and/or assessing response during treatment. Further translational studies focusing on side effects, drug resistance, and combination of traditional chemotherapeutic drugs, NSAIDs and anti-lymphangiogenic therapies may improve the outcome of current cancer treatment regimes.
